# Religion, Politics, and Vaccines: Elaborating the Integrative Public Policy Acceptance (IPAC) Framework Through HPV Vaccine Program Acceptance Among Religious Leaders in Bangladesh

**DOI:** 10.34172/ijhpm.9179

**Published:** 2026-01-07

**Authors:** Md Towhidur Rahman, Sultan Mahmood, Katie Attwell

**Affiliations:** ^1^Government of Bangladesh, Ministry of Public Administration, Dhaka, Bangladesh.; ^2^VaxPolLab, School of Social Sciences, The University of Western Australia, Perth, WA, Australia.; ^3^ShuHaRi Health, Dhaka, Bangladesh.

**Keywords:** Cervical Cancer, HPV Vaccine, Vaccine Hesitancy, Bangladesh, Muslim Religious Leaders, Political Consensus

## Abstract

**Background:**

In October 2023, Bangladesh introduced a free, single-dose human papillomavirus (HPV) vaccine for girls aged 9–14 through its national vaccination program to prevent cervical cancer, the second most common cancer among Bangladeshi females, caused by the HPV. Although vaccine hesitancy was not a significant issue before the COVID-19 pandemic, experiences from that pandemic and global literature suggest that the population’s uptake of this vaccine may face barriers due to concerns related to reproductive health, fertility, and cultural and religious beliefs. This is particularly relevant in a country where Islam is the state religion, 91% of the population is Muslim, and religious leaders hold significant influence over public opinion.

**Methods:**

Building upon the recently developed Integrative Public Policy Acceptance (IPAC) framework, this qualitative study explores the factors shaping religious leaders’ support for the HPV vaccine informing their potential role in promoting it. Semi-structured interviews with leaders from Bangladesh’s five main Islamic traditions were thematically analysed using NVivo 14 with inductive and deductive coding.

**Results:**

Islamic religious leaders’ varying support for HPV vaccinations in Bangladesh was influenced by their limited awareness of cervical cancer, as well as their religious and social concerns about ingredients, side effects and a fear of promoting promiscuity. Political ideologies also played a significant role, as leaders were less supportive of the program when they perceived the government as ideologically opposed to the beliefs or practices of their specific religious tradition.

**Conclusion:**

The study’s contribution to the IPAC framework highlights the importance of political consensus in policy acceptance, explaining how partisanship and ideological differences impact public policy compliance. The findings underscore the need for health systems in Muslim majority countries to engage with religious authorities, build political inclusivity and consensus, and align health policies with religious and cultural values.

## Introduction

**Key Messages**

**Implications for policy makers**

- Religious leaders in Bangladesh support vaccines in general, but become increasingly sceptical—particularly regarding ingredients, efficacy, country of origin and potential side effects—when the vaccine concerns the female reductive system or is linked to a sexually transmitted infection (STI).
- Political suppression, unequal attention to different political groups by the government, distrust in the government due to lack of transparency in democratic decisions, and divergent political ideologies can hinder non-political agendas such as vaccination programs; therefore, political consensus can enhance adherence.
- Engaging religious leaders from diverse Islamic traditions—including those beyond government-affiliated institutions like the Islamic Foundation—early in the policy-making process, and providing targeted, culturally sensitive education on cervical cancer and the human papillomavirus (HPV) vaccine, can strengthen their support for vaccination by reducing partisan distrust.

**Implications for the public**

In a country like Bangladesh, the public listens to health-related messages from the government and other influential sources—particularly religious leaders—and makes decisions accordingly. The public is also part of the political community and plays a role in pushing the government and institutions towards transparency, democracy, and good practices. This multifaceted feedback loop shapes government actions from time to time, which may, in turn, influence the perceptions of both religious leaders and the public. In such a context, this study helps readers understand the underlying motivations behind religious leaders’ health-related sermons and future messages regarding Bangladesh’s human papillomavirus (HPV) vaccination program—whether they are supportive or hesitant. Public awareness of vaccine-preventable diseases, government campaigns, and religious leaders’ perceptions, can optimise the success of the vaccination program.

Cervical cancer is the second most prevalent cancer among women in Bangladesh, placing 64 million women aged 15 and older at risk, with 8268 new cases and 4971 deaths reported annually.^1^ The Government of Bangladesh launched a nationwide human papillomavirus (HPV) vaccination campaign in October 2023, providing a free single-dose vaccine to all girls aged 9 to 14 years to prevent the infection that causes most cervical cancers.^2^ However, following the COVID-19 pandemic and vaccination program, uptake of HPV vaccination faces challenges from emergent religious opposition to COVID-19 and other vaccines. This adds to the existing challenges of the religious optics of vaccinating adolescents against a sexually transmissible disease, as experienced in many other countries.^3-12^ In such a context, it is important to consider the perspectives of religious leaders of the various Islamic traditions in Bangladesh, and to assess the support or opposition that they bring to government efforts to prevent cervical cancer in Bangladesh.

## Background

 Vaccine hesitancy or refusal was a minimal problem for immunisation efforts in Bangladesh prior to the COVID-19 pandemic. Bangladesh’s Expanded Program on Immunisation has achieved remarkable success in attaining extensive coverage.^13^ This successful implementation of childhood vaccination may indicate that the Bangladeshi population are not generally vaccine hesitant. However, the COVID-19 pandemic highlighted a different scenario. With the introduction of the COVID-19 vaccine, people began to express hesitancy. Several studies highlighted high levels of hesitancy in Bangladesh driven by rumours, misinformation, religious beliefs, the country of development/manufacture, vaccine ingredients and side effects, and political beliefs.^14,15^ News reports and research articles highlighted that religious leaders in Southeast Asian countries, including Bangladesh, influenced their followers not to take the COVID-19 vaccine, by spreading rumours, spreading misinformation and conspiracy theories claiming that the virus was not dangerous and the vaccine was not religiously permissible and was part of a conspiracy by foreign nations.^16-20^ Nevertheless, many other religious leaders actively joined the effort to combat the pandemic by using mosque loudspeakers to disseminate essential public health messages to prevent COVID-19.^21^ These differences in whether religious leaders support or undermine immunisation, as highlighted in prior studies, show that when authorities actively and respectfully involve them, these trusted figures help dispel misconceptions and promote vaccine acceptance.^22,23^ By contrast, a lack of engagement can lead to doctrinal objections, concerns about vaccine ingredients, and the spread of misinformation that undermines public health goals.^24-26^

 Bangladesh is the 8th most populous country in the world, with a population of 170 million, 91.04% of whom are Muslim.^27^ Religion holds immense influence in daily life, with Imams (mosque prayer leaders), religious speakers (who address large gatherings), and religious leaders exerting considerable sway over the populace. Many decisions, including those related to healthcare, are influenced by these religious figures.^28,29^ Notably, there are ideological divisions within religious groups (within Islam), meaning that decisions supported by one ideology may be viewed as fallacious by another.^30^

 Previous research has identified several common barriers to HPV vaccination across different cultural contexts, including concerns about fertility, fears that the vaccine may encourage promiscuity or sexual disinhibition, and doubts about vaccine ingredients and safety.^3-6,9,31,32^ These concerns have been observed globally, across both Muslim and non-Muslim populations.^4,9-11,33^ However, in some communities, including Muslim ones, such anxieties are often framed through religious or moral reasoning relating to modesty, sexual ethics, or divine will.^7,8,10-12,34-40^ Understanding how these globally shared concerns manifest within the Bangladeshi Islamic context is therefore essential to designing communication strategies that resonate with local religious values and promote HPV vaccine uptake effectively.

 Since Bangladesh’s mass vaccination program for HPV is new, no existing studies assess religious leaders’ perceptions on preventing sexually transmitted infections(STIs) and HPV through medical interventions. Accordingly, this study aims to assess the policy acceptance of religious leaders in Bangladesh regarding the HPV vaccination program, exploring beliefs regarding diseases and vaccination, political philosophies and affiliations, and overall worldviews. These perspectives are analysed using the Integrative Public Policy Acceptance (IPAC) framework to assess their potential role in HPV vaccination by explaining the aforementioned factors that impact their willingness to support, promote and participate in the vaccination program.^41^

## Theoretical Framework

 To assess citizens’ likelihood of accepting a policy, Grelle and Hofmann proposed the IPAC framework.^41^ They argue that citizens’ problem awareness (ie, knowing, understanding, and recognising the problem) regarding a particular issue in society influences policy acceptance, which can further impact those individuals’ compliance with policies designed to address that problem. Citizens’ desire for governmental support or intervention to solve that specific problem then mediates the relationship between problem awareness and policy acceptance. Another contributing factor is the support-seeking characteristics of citizens (ie, control attribution, responsibility attribution, trust in the government, and value fit). According to the framework, Control and Responsibility Attribution refers to whether individuals see a problem as within their own control or as the government’s responsibility, with higher external attribution increasing the desire for governmental support. Trust in Government influences this relationship by shaping individuals’ confidence that the government will act in their best interest, thereby strengthening the link between problem awareness and support for intervention. Additionally, Value Fit plays a crucial role in policy acceptance, as individuals are more likely to support government action when policies align with their personal values and political beliefs. These features also determine the link between problem awareness and the desire for governmental support. Finally, Perceived Qualities of proposed policy solutions to address a specific problem (ie, effectiveness, intrusiveness, transparency, fairness, cost, and benefit) also play a role in policy acceptance.

**Figure 1:**
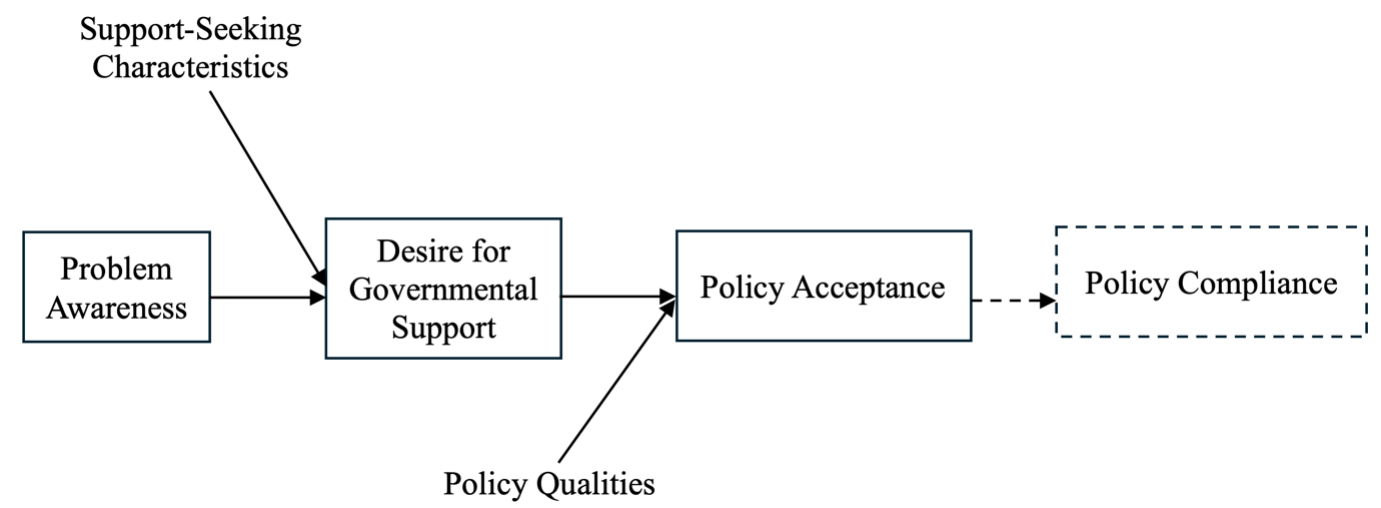
The Integrative Public Policy Acceptance Framework.^41^

Figure 1 presents the visual expression of the IPAC framework proposed by Grelle and Hofmann.

 The IPAC framework is useful for considering the perceptions of religious leaders in Bangladesh regarding HPV vaccination to prevent cervical cancer and how they may subsequently endorse or subvert the program. It guides analysis of their individual dispositions, personal attitudes, and beliefs regarding whether and how they (*a*) understand the relationship between cervical cancer, HPV, and the new vaccination program, (*b*) see cervical cancer as a serious problem, (*c*) support government intervention, (*d*) believe the government should intervene to prevent cervical cancer, and (*e*) hold particular positive or negative views about the vaccine and the vaccination program.

 However, “compliance” in the context of religious leaders means something different than the direct policy compliance of the original framework. If we were considering acceptance of the vaccine amongst the general population in Bangladesh, we would regard “policy compliance” as vaccine uptake. However, our interest in religious leaders is not as individual decision-makers regarding the vaccination of women and girls in their families (though this may be relevant). Instead, we are considering the special role that religious leaders play as advocates (or opponents) of vaccination amongst the communities they serve, disseminating or amplifying messages regarding the safety and necessity of vaccination. Accordingly, it makes more sense to replace “compliance” in the original framework with “policy support.”

## Methods

###  Study Overview

 In this exploratory qualitative study, we sought to capture the diversity of the main religious traditions in Bangladesh’s complex religious landscape. Spanning the religious landscape required purposive sampling from different traditions distinguished by (*a*) education and (*b*) institutional/organisational affiliation. Aliya and Qoumi are the two main madrasah education systems in Bangladesh. From its inception, the Aliya system has been state-regulated and publicly funded, designed to produce scholars equipped with both religious and general education.^42^ In contrast, the Qoumi system evolved independently as part of Muslim responses to colonial education policies, developing outside government control and sustained through community donations and religious endowments.^43,44^ While Aliya education follows state guided curriculum and examination procedures, Qoumi madrasahs focus on a Dars-i-Nizami framework emphasizing the Quran, Hadith, and classical Islamic texts, taught through traditional, teacher-centered pedagogy.^45^ Socially, Aliya madrasahs function as a bridge between religious and mainstream education, whereas Qoumi institutions continue to play central roles in community religious leadership and social welfare.^44^

 Considering organisational affiliation, we have also drawn from three major traditions: Tabligh, Islamic Foundation, and Sufism. The Islamic Foundation is an autonomous government organisation based in the capital, Dhaka, functioning through an extensive network of grassroots-level offices, delivering imam training, and operating training academies as well as mosque-based Islamic education at the grassroots level.^46^ Tablighi Jamaat (Society of Preachers), a non-political and highly influential 20th-century international religious movement, was founded on Qoumi ideology with origins in the revivalist tradition of the Deobandi school to revive declining Islamic moral values.^47,48^ Sufismin Bangladesh comprises multiple lineages centered on *pirs (spiritual teachers), Khanqahs (hospices) and dargahs (mausoleums), *and these actors play significant roles in popular spirituality, social welfare, and local religious leadership.^49,50^ Sufism does not represent a unified organisational structure nor a single group, but a plurality of orders that link Muslim communities across South, Central and Western Asia.^51,52^ Religious leaders educated in Aliya, Qoumi or other system can affiliate with any of these religious organisations.

 By sampling across both educational system and organisation, we ensured participation from all major religious segments in Bangladesh. Our study design and sample size meant that we could draw only tentative conclusions about the impact of the *differences* between these traditions. Nevertheless, we were keen to contextualise the perspectives of participants within their specific religious affiliation, as well as consider commonalities and differences across the entire sample.

###  Participants and Recruitment

 To enact our proposed recruitment strategy of interviewing two religious leaders from each of the five groups as described above (two based on their educational background – Aliya and Qoumi; and three based on organisational affiliation – Islamic Foundation, Tabligh, and Sufism), a “fit-to-purpose”^53^ and snowball sampling practice was utilised. The lead author recruited all participants. The first round (five participants) was reached using that researcher’s personal and professional networks. Subsequently, snowballing was used to reach the remaining five participants. All participants were contacted directly by telephone and email by the lead author and asked to participate. One religious leader initially consented to participate but later stopped responding. To fill this gap, another participants was recruited using the snowball sampling method.

###  Data Collection

 A semi-structured interview guide was designed for this study based on the available previous research on HPV vaccination acceptance across various geographical locations and religions, especially those applicable to Bangladesh and Islam. We also drew from indicative questions from the World Health Organization’s (WHO’s) “Behavioural and Social Drivers of Vaccination” guide.^54^ Additionally, since cervical cancer is a disease caused by persistent infection with HPV (an STI), we used STI as a proxy to assess participants’ broader perceptions of cervical cancer, HPV, and their willingness to promote vaccination. This approach was adopted because HPV is widely recognised in both international and governmental documents as a common STI,^55^ and the term “HPV vaccine” may lead religious leaders to associate it with sexual activity, potentially influencing their attitudes. We sought to be transparent about this so that we could understand how religious leaders responded.

 The interview guide covered participants’ educational backgrounds, organisational affiliations and perceived influence on their congregation; awareness and understanding of cervical cancer, HPV, and vaccination; religious beliefs and attitudes toward vaccination and sexuality; perceptions of broad community attitudes regarding communication with their congregation, gender based social perspectives and response to government instructions; roles in public health communication; past experiences of misinformation regarding vaccination, prevention and treatment of diseases; perceptions of engagement with government initiatives; and ideas for policy recommendations.

 We discussed numerous times throughout our design process that participants may not know much about HPV or cervical cancer, and how to give them enough information so that they could then assess and tell us their thoughts. This raised potential risks of us biasing their reflections as we explained this information. We further considered the potential real-world consequences of this: that in seeking to provide enough information in order for the participant to be able to answer our research questions, we might inadvertently raise controversies around sexuality and morality that would influence the ways that the religious leaders would subsequently talk to their communities. To mitigate these concerns, we developed an information pack based on a WHO factsheet. Our Participant Information Form, which we sent before the interview, included details about cervical cancer (name, cause, transmission, and statistics). At the interview’s conclusion, we asked if participants wanted more information about the disease or research. Two participants showed interest, so we briefed them verbally and provided awareness flyers created by Bangladesh government agencies.

 We iteratively adjusted the question guide over the course of the interviews to further probe emergent findings regarding international funding for the vaccine, doubts about ingredients and geopolitical conspiracies, and the current state of childhood vaccination. Finally, our question guide was not informed by the IPAC framework, which we only adopted at analysis stage. However, our focus on the leaders’ acceptance and endorsement of the vaccination program meant that we collected data across all relevant domains, as well as developing new ones (elaborated below).

 Along with the Participant Information Form, a Participant Consent Form was sent to the participants via email and WhatsApp. Participants then provided verbal consent at the beginning of each interview, as appropriate for a country with limited connectivity for sending electronic documents.

 Interviews were conducted online in the Bengali language, the mother tongue of the lead author and the participants. The lead author transcribed the interviews in full in Bengali, then manually translated the findings into English. Analysis was conducted in English in collaboration with the senior author.

###  Data Analysis

 NVivo 14 was used for data analysis and coding, initially employing an inductive approach followed by deductive coding using the IPAC framework.^56^ Deductive codes were used to assess participants’ awareness of the problem (knowledge and understanding, recognition), support-seeking characteristics (control attribution, responsibility attribution, trust in government, and value fit), and perceived policy qualities (effectiveness, intrusiveness, transparency, and costs and benefits), as per the IPAC framework.

## Results

###  Demographic Information of the Participants

 Brief demographic information about the participants (collected during the interviews) is presented in Table. Participants had received education from multiple systems and were affiliated with multiple organizations; categorising the main affiliation made it easier to ensure the representation of all prominent streams of religious practices in Bangladesh.

**Table:** Demographics of the Participants

**Participant Code**	**Affiliation**		**Occupation**	**Age (y)**	**Years of Experience**
	**Educational**	**Organisational**			
Participant 1	Aliya	Islamic Foundation	Fulltime Imam	>50	>20
Participant 2	Aliya	Sufism	Fulltime Imam	40-50	>20
Participant 3	Hafiz*, Aliya	Islamic Foundation	Fulltime Imam	30-40	<10
Participant 4	Hafiz*, Aliya, Qoumi	Tabligh	Fulltime Imam	40-50	>20
Participant 5	Qoumi	Islamic Foundation	Imam trainer	40-50	10-20
Participant 6	Informal Islamic Education	Tabligh	Religious speaker	40-50	>20
Participant 7	Hafiz*, Qoumi	Independent	Religious speaker	30-40	<10
Participant 8	Aliya	Sufism	Religious speaker	30-40	<10
Participant 9	Informal Islamic Education	Tabligh	Religious speaker	40-50	>20
Participant 10	Aliya	Independent	Teacher	>50	>20

* Memorize the Holy Quran (can be from either Aliya or Qoumi education).

###  Awareness and Recognition of Cervical Cancer and STIs as Public Health Problem

 Amongst the religious leaders we interviewed, cervical cancer awareness was very limited. Although they had previously heard of the disease, they were unaware of its potential cause and could only associate it with a *female disease*. None were familiar with the term “HPV” indicating a lack of awareness about causes, carriers, and transmission. Additionally, at the time of the interview (December 2023 to March 2024), none were aware of the government mass vaccination program already underway providing free HPV vaccination.

 Regarding awareness of STIs beyond cervical cancer, participants reported an alarming rate of various STIs in Bangladesh. They attributed the prevalence to insufficient morality and religious practice, co-education, family violence, the spread of technological advancements that provide access to illicit web content, and insufficient oversight of prostitution.

 Most participants viewed cervical cancer as a threat to women in Bangladesh but struggled to explain why, other than that“*cancer sounds fatal*” (P1, P5, P6, P7). A minority thought that neither cervical cancer nor STIs are significant threats in Bangladesh, since it is a Muslim country where most people “*strictly practice religion.”* They saw those “*diseases as potential risks [only] in Western countries, where Islamic lifestyles are not followed” *(P8).

 In their recognition and framing of the problem, half of the participants explicitly recognised the social and medical vulnerability of women seeking information and treatment for cervical cancer and STIs. They identified this vulnerability as a main factor contributing to their ailments from these diseases. They pointed out how the “*stigma associated with the disease”* drives women to “*endure it silently for a long time before seeking help” (*P1), which affects survival.

 No participant had discussed cervical cancer, STIs, or any related health issues in their religious speeches or sermons. They attributed this absence to both “*embarrassment in discussing such issues*” and “*a lack of knowledge*” (P5). However, in reflexively considering their influence to motivate people to protect their health, as well as the prevalence and impact of these diseases on victims, participants felt “*it is important not to keep it hidden”* (P1)and expressed the importance of discussing these issues in religious talks.

###  Support Seeking Characteristics

####  Responsibility and Control Attribution

 Specific questions were asked regarding whether participants felt that medical interventions, such as vaccination, are necessary to control cervical cancer. Most believed that strictly adhering to religious values could prevent cervical cancer and STIs, viewing moral character and religious commitment as more effective than vaccines. Nevertheless, several expressed support towards recommending HPV vaccine to their daughters or relatives once it was raised in the interview. As the discussion progressed and the participants learned that HPV can be transmitted through sexual acts, most then expressed further concerns regarding the vaccine. For example, Participant 3 said:

 “*…if I can research or gather information from acquaintances that this disease will not occur if one maintains good moral character, then my enthusiasm for getting [my daughter] vaccinated decreases…. In my opinion, everyone in our society should be disciplined regarding their own sexuality. We don’t have any alternative to this….” *

 Most participants believed that religious practices can control the disease, and saw themselves as responsible for promoting religious teachings to prevent cervical cancer and other STIs.

####  Value Fit 

 Questions were asked to understand whether participants perceived vaccination against cervical cancer and STIs as aligned with their religious and cultural values. Assessing these values is important, as beliefs that vaccine ingredients are Haram, disease prevention opposes divine will, illnesses are divine tests, or STIs regulate behaviour can reduce support for vaccination programs.

 Most participants believed that diseases, including cervical cancer or STIs, are either the result of sin, a curse, or a test from the Almighty, and that STIs provide God-given safeguards against immoral behaviour by discouraging people from straying beyond religiously legitimate relationships. They thought that informing adolescents about the effectiveness and purpose of the HPV vaccine might lead them toward a lifestyle that is not permitted in Islam, and they canvassed deliberately repressing this information. Participant 6 expressed that “*it is not necessary to explain to girls why this vaccine is given to them*.” Others suggested addressing this perceived concern through additional interventions: “*While administering the vaccine, parallel advocacy for leading a religious life should be conducted*” (P4).

 A minority took a different view on God-given safeguards. Countering the idea that people will refrain from wrongdoing due to the fear of contracting diseases or that preventing diseases through vaccination may promote such wrongdoings (promiscuity), these participants argued that neither diseases nor vaccines influence moral behaviour; rather it is a more over-arching fear of the Almighty that prevents immoral behaviour. These participants supported vaccination to prevent STIs: *“Promoting the vaccine would raise awareness of the dangers of these diseases, which would eventually encourage personal responsibility*” (P10).

####  Trust in Government and Political Attitude

 We assessed participants’ political attitudes and their trust in the government and other relevant institutions as the provider of the vaccination program from three perspectives. First, do they believe the government treats all religious groups equally when formulating or implementing a non-political agenda (such as public health or immunisation policy), given that religious groups and the government may hold to different political ideologies? Second, if they believe the government treats groups unequally, how does this influence policy implementation, particularly in terms of complying with government instructions by engaging their followers? Third, what are their political attitudes regarding funding from overseas governments or organisations, and how do they perceive international politics?

 Participants who were not affiliated with the government controlled Islamic Foundation generally believed that the government does not treat every religious group equally when evaluating opinions in policy discussions. “*The government always emphasises the opinions of religious leaders and scholars who are affiliated with the Islamic Foundation, and they try to implement its agendas through the leaders and imams from the foundation, and that is why the results are not widespread in our society*” (P4).

 Some held a slightly different view, suggesting that the government *tries* to evaluate and engage all religious groups equally, but that *realpolitik *constraints prevent this. They pointed out that the government builds relationships based on “*future polling calculations and the ability of religious groups to exert pressure on the government*” (P8). These participants believed that groups perceived by the government as non-threatening received fairer treatment.

 Although some participants expressed high trust for government regarding health issues and social initiatives, differences regarding political ideologies and political suppression appeared to influence religious leaders’ compliance with government instructions on health and social initiatives more generally. For example, participants noted receiving government sermon instructions regarding social and health issues but admitted low trust and compliance. Many felt these directives masked political agendas, provoking scepticism among religious leaders and congregation. In this regard, participant 4 stated:

 “*Despite the government sometimes speaking the truth, due to widespread discontent, political differences, or a lack of trust in the government for various reasons, people may not believe the government… The government propagates the information in its own way, and people tend to resist accepting it.”*

 Such political discontent led participants to merely read out sermon instructions in front of their congregation without discussing them.

 Participants’ political attitudes towards foreign funding for vaccination were divided. Half were concerned about such funding and collaborations, while the rest saw benefits for humankind. Critics expressed concerns about nefarious agendas to depopulate the Muslim community and weaken the country’s geopolitical and economic interests and negotiation capabilities in the long run. They worried that reliance on donor funds might constrain governments from making beneficial decisions for the population in the future, and that Bangladesh would be dependent on foreign donor nations. These thoughts reflect their historical perspectives on the relationship between Muslim and non-Muslim communities, as well as the ongoing crises and wars in the Middle East. In this regard, participant 4 stated:

 “*Generally, when the government or some international organisation provides us with any free vaccine, we tend to have significant doubts about it. Is there an economic, political, or international political interest behind such initiatives? Because those non-Muslim communities that discriminate against Muslims worldwide spend billions of dollars to provide us with vaccines—what is the real intention behind it?”*

 The notion of something ‘free’ from foreign countries intensified participants’ distrust in vaccines, especially those provided at no cost by foreign nations or organisations. This reflected participants’ scepticism toward foreign donors, international organisations, and the government’s failure to inform them about the efficacy of vaccines and the facts regarding international collaborations to combat infectious diseases.

###  Perceived Policy Qualities

####  Benefits and Potential Contribution of Vaccines 

 Almost all participants stated that vaccines are generally effective in preventing diseases, and that there is nothing wrong with taking vaccines and seeking medical treatment from an Islamic perspective. However, according to their religious beliefs, the effectiveness of any medical intervention, including vaccines, depends on the will of the Almighty, who alone has the power to heal. Therefore, they advise their congregants “*to rely on Allah first, pray for recovery, cite relevant Surahs (verses from the Holy Quran), make charitable contributions to the poor, and then, as a last resort, seek medical interventions*” (P6). Participants appeared to hold this view in a somewhat contradictory manner alongside their generally strong support for vaccination as a concept and practice, as discussed earlier. However, when it came to HPV vaccination, they expressed concerns about side effects, as discussed below.

####  Transparency and Intrusiveness of Vaccine Policies 

 Participants felt that vaccines, their promotion, and related policies lack transparency. Concerns regarding ingredients were sometimes less about safety and more about religious legitimacy (ie, the ingredients are not clear, they are written in scientific or chemical terms, there is no halal certification for the vaccines, and no agency in Bangladesh certifies their religious legitimacy). However, participants also noted that vaccine promotion lacks transparency concerning potential side effects. In the case of HPV vaccination, they were particularly concerned about fertility, since the disease involves the female reproductive organs. Other policy related concerns included age and sex eligibility (eg, why some vaccines are given only to females and not males, and why particular ages are targeted), and vaccine efficacy. A few participants (P6, P9, and P10) explained that “*any ingredients can be used, even if those are religiously illegitimate, to save lives through medicines and vaccines*” (P9) but most shared doubts about the ingredients of foreign-produced vaccines, which may relate to their distrust in international collaboration, as discussed above. Participant 7 stated:

 “*Since the vaccines come from outside the country, there is doubt among people about the ingredients and whether they are properly informed about the side effects of these vaccines.”*

 Participants agreed that vaccine mandates encourage vaccinations effectively, citing the COVID-19 vaccine requirement for travel to Mecca as an example. However, some religious leaders viewed mandates as intrusive, asserting that vaccination is an individual right that should not be coerced by the government or any other entity. They argued that “*it is an individual’s right to seek medical treatment and take vaccines*,” and “*neither religion nor laws should intrude on this choice*” (P3, P5, P6). Participants’ opposition to mandates may align with their religious beliefs: strong believers might refuse vaccines, relying instead on the will of the Almighty, as mentioned earlier.

## Discussion

 This exploratory study considered Islamic religious leaders’ attitudes towards the new HPV vaccination program in Bangladesh for young and adolescent girls, and how this may affect their potential support or opposition for the program as they discuss related issues with their followers. Our novel findings identified limited awareness of cervical cancer amongst religious leaders, as well as significant religious and social concerns about vaccine ingredients, side effects, and a fear of promoting promiscuity. The more participants learned about HPV and its causes and prevention during the interview, the less supportive some became of the program. This was something we sought to mitigate but could not avoid. We found that political ideologies and beliefs also played a significant role, as participants tended to be less supportive of the vaccination program when they perceived the government as ideologically opposed to their beliefs or practices and felt suppressed or neglected due to such differences.

 In the sections to follow, we consider the implications of our findings in the context of the IPAC framework. We focus on the desire for governmental support influenced by problem awareness and support-seeking characteristics, leaving out how policy quality influences policy acceptance. We found that participants’ perceptions of vaccine effectiveness, policy intrusiveness, and transparency resonated more with their perceived values, which fall under support-seeking characteristics. Additionally, we examine how participants’ desire for governmental support was influenced by their political ideology and the existing political environment of the country, as this emerged as a distinct feature in our study findings.

###  Desire for Governmental Support Influenced by Problem Awareness

 The desire for government support is a key determinant of policy acceptance. The IPAC framework highlights that a greater awareness of a problem increases the desire for government support, ultimately enhancing policy acceptance.^41^

 Our findings show that religious leaders in Bangladesh are not sufficiently aware of cervical cancer to feel a strong urge to promote the HPV vaccine. Although no previous studies specifically assess cervical cancer awareness among religious leaders in Bangladesh, research on parents, university students, medical professionals, and various male and female cohorts shows similarly low awareness. Most people recognise the disease by name but lack knowledge of its cause, transmission, prevention, or the HPV vaccine’s efficacy and eligibility.^57-60^ Along with this low level of awareness, religious leaders often misattribute cervical cancer and STIs to coeducation (mixed-sex schooling) and technological advancements, such as easy access to illicit content, potentially diverting public attention from the real causes.

 Religious leaders in our study generally supported vaccinations and medical interventions. However, while our participants initially declared support for vaccines to prevent diseases, some walked this back for HPV when they learned that the disease is related to an STI. This hesitancy may be influenced by concerns about sexual disinhibition or the perception that vaccination could promote promiscuity,^35^ the cultural conservativeness of Muslim societies regarding discussion of sexual taboos and disclosing other’s secrets,^61^ or the belief that religious communities are largely free from such diseases, which can contribute to underreporting.^62,63^ Similar hesitancy has been observed among Christian and other faith communities, where concerns that promoting the HPV vaccine might be perceived as condoning premarital or extramarital sexual activity have shaped attitudes toward HPV immunization.^25,64-67^ These tendencies may reduce religious leaders’ desire for governmental support (ie, for the government to roll out a mass vaccination program for HPV), ultimately making it more challenging for authorities to involve them in promoting uptake. These findings suggest that an approach of discussing HPV vaccine as a vaccine against a virus and addressing understanding of how immunisation works (ie, normalizing the HPV vaccine alongside other vaccinations and under-playing the STI angle) is likely to be a more productive framing for bringing religious leaders on broad as supporters. However, we worry that they may still react negatively as and when the STI connection becomes apparent to them, even if this occurs much later. Moreover, if they perceive that this connection was deliberately obscured, this will have an impact on the perceived legitimacy of the campaign and the institutions involved, particularly in the context of the fragile political trust we elaborate.

 Our participants identified the social stigma faced by females when seeking medical support or information regarding STIs and HPV. The lack of information and the embarrassment surrounding such discussions align with findings from previous studies.^7,8,10-12^ In this present study, religious leaders agreed that low awareness (both among the population and themselves), along with people’s tendency to keep these diseases and discussions hidden or taboo, puts vulnerable women at risk. They also expressed a desire to learn more about these diseases to motivate people, as they saw themselves as influential in shaping public behaviour. Their lack of awareness of government initiatives on HPV vaccination, as well as the causes and prevention of cervical cancer, indicated that existing information provision strategies on these issues are not effective and need to be redesigned.

###  Desire for Governmental Support Influenced by Support-Seeking Characteristics

 Insights from the IPAC framework suggest that the willingness of religious leaders to seek government support for preventing cervical cancer through the HPV vaccination program will be influenced by several factors. First, whether they believe they have control over the issue and see themselves as responsible for addressing it or whether they feel government intervention is necessary (control and responsibility attribution). Second, they must also believe that the government will act in alignment with their values and the norms they uphold. Lastly, their trust in the government—specifically, the belief that government intervention will be carried out in a manner that ensures their welfare—will also play a crucial role.^41^

 Regarding control and responsibility attribution, our participants believed that STIs are the result of God’s wrath due to the prevalence of illicit and sinful sexual acts in society, a finding consistent with previous studies.^61,63^ This philosophy leads religious leaders to attribute illicit sexual behaviour as the sole cause and control factor for preventing STIs. They view STIs as a divinely gifted safeguard to regulate human behaviour and keep society on a religious path, and they see themselves as responsible for promoting these religious teachings among their followers. Previous studies show that when societies uphold the requirements of abstinence before marriage and complete sexual fidelity, the perceived purity of women leads people to become reluctant to seek vaccines related to STIs.^64,68^ Prior research suggests that women in conservative Muslim societies face obstacles in seeking vaccination or even treatment for HPV and other STIs because of the fear of being labelled as promiscuous.^10,69^ This normative notion of behaviour, judged by religiosity, further defines the perceived purity of women (abstinence until marriage) and acceptable relationships (only marital relations). Our participants’ views on control and responsibility attribution regarding the prevention of cervical cancer through vaccination reflect a lack of desire to seek governmental support for addressing the HPV problem.

 Findings from the value fit attribution reveal three perspectives. First, some participants believed that the introduction of the HPV vaccine may not align with existing values because they were unsure about the vaccine’s ingredients and preparation process, which they feared might not comply with religious legitimacy. Second, some participants worried that the vaccine might encourage promiscuity, which went against religious and social norms. Lastly (and more positively), some religious leaders thought that the vaccine aligned with religious values and social norms, arguing that for medical purposes, any ingredient, even a “Haram” one, could be used as a life-saving medicine. They believed promoting the vaccine would raise awareness about the causes of STIs, ultimately benefiting the population.

 The first two perspectives align with previous studies.^9-11,32,33,39,40,70-73^ The third perspective, in which religious leaders express support for the HPV vaccine, reflects a shift toward viewing themselves as enablers. Previous research suggests that social influencers—especially religious leaders—often feel motivated to promote vaccination among their followers when certain conditions are met. Leaders will encourage vaccination when they perceive this activity as part of their moral or social responsibility,^22,74,75^ when they observe recognition of their peers or groups by health authorities,^76^ when they recognise the demonstrated effectiveness of the vaccine or an emerging public-health necessity,^77^ and when the intervention is framed as consistent with their religious teachings, ethical duties, and broader social norms.^22,74,75^ Consistent with these findings, some participants in this study expressed appreciation for government-led cervical cancer prevention initiatives through vaccination programs. This group can be effectively targeted in future policy design, as the relationship between acceptance and compliance can sometimes be obscure ie, even with low awareness and acceptance, people may comply with a policy due to strong societal norms and peer influence, prompting opposing groups to become enablers because their peers support what they initially opposed.^41^

 Some of our participants shared beliefs regarding an individual’s right to reject the vaccine based on their faith and reliance on God. This aligns with previous literature. Other studies have found that vaccine hesitancy can stem from the belief that diseases originate from God as a matter of fate or destiny, and individuals have no control over the will of the Almighty.^24,78-84^ This perspective can potentially divide the congregation in the eyes of religious leaders into those who truly trust and rely on God and those who do not. Such division may discourage some people from opting for vaccines, as doing so might be seen as a sign of weak faith in God.^24,25,85^

###  Desire for Governmental Support Influenced by Political Ideologies

 The IPAC framework suggests that “personal and political attitudes and beliefs or political orientation” and “trust in government” play important roles in increasing the likelihood of people’s desire for governmental support.^41^ While discussing “personal and political attitude” and “political orientation,” Grelle and Hofmann focus more on liberal versus conservative political beliefs. In discussing “trust in government,” they emphasize people’s trust that the government will act in ways that promote societal welfare. These framings may reflect the way that politics operates in stable, high-income, Western countries, but they do not adequately reflect the role of politics in non-Western societies.

 Beyond “political orientation” and “trust in government,” we argue that political suppression by the state, public discontent, and conflicts between the state and stakeholders—in other words, a lack of political consensus—foster significant partisanship among social, religious, and political groups. This, in turn, affects policy acceptance, even when the policy pertains to non-partisan issues (eg, health). Previous literature also supports this view: political polarization influences people’s willingness to follow government policy decisions,^86^ creates discrimination against individuals with differing political ideologies,^87^ and fosters a culture of distrust between parties. We focus on the factors that influence political consensus; particularly how inequitable treatment by the government of different societal groups can impact it. Such inequity may result from political suppression by the government. If the government perceives a specific social or political group as a threat, the resulting actions may lead to further political discontent and decreased compliance with government policies by those groups.

 Aligning the idea of “Political Consensus” with the IPAC framework, this study identifies four key factors that can influence religious leaders’ willingness to seek governmental support for an issue (as per the framework)—or, in the terminology of our study—how they may respond to a government initiative, and in particular what they may do if or when the government seeks their involvement in vaccine promotion. These factors are: (1) how they perceive the government evaluates existing religious groups; (2) why the government may or may not treat all religious groups equally; (3) how differences in political ideologies may discourage policy support or compliance, even if the policy benefits society; and (4) how religious leaders view international and geopolitical issues.

 The first three factors are interrelated. Our findings show that religious leaders do not believe the government evaluates or treats all religious groups equally. Participants believe that the government assesses religious leaders based on (*i*) vote-centric political calculations, (*ii*) their political ideologies and movements, particularly whether they pose a challenge to the government, (*iii*) their ability to exert pressure, and (*iv*) their affiliation with the Islamic Foundation or the ruling party’s religious political branches or alliances. This perception of disaggregation creates polarisation or partisanship among religious leaders, which in turn influences their policy acceptance and compliance, as well as their willingness to seek governmental support. Additionally, when the government attempts to engage a portion of religious leaders through affiliations with its organisations (such as Islamic Foundation), political polarisation increases, leading some religious leaders to distrust others who are aligned with the government. These findings resonate with previous studies, which show that people with greater trust in the government are more likely to comply with public health-related restrictions and instructions. This trust also deepens based on the image of the head of government among the target population.^88-91^

 At the time this study was conducted, the then Prime Minister, Sheikh Hasina, was widely accused of violating human rights, undermining democratic practices, rigging elections, enabling institutional corruption, and harassing opposition figures, including religious and political leaders. She later resigned and fled the country following a people’s revolution on August 5th after 16 years of successive rule.^92,93^ This political context helps to explain why religious leaders expressed opposition even to beneficial government initiatives. It underscores the importance of democratic practices and trustworthy institutions across all sectors of government to encourage compliance with HPV vaccination efforts and other public policies. Previous studies from different regions of the world show that high levels of trust in government consistently correlate with increased vaccine uptake and reduced hesitancy, while distrust remains a primary driver of noncompliance.^94-100^

**Figure 2:**
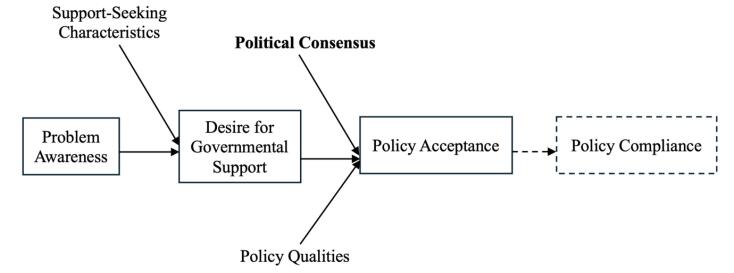
Revised Integrative Public Policy Acceptance Framework (Political Consensus as factor influencing Policy Acceptance).

 Taking the findings into consideration, this study identifies additional distinct dimensions of government trust and political orientation in the IPAC framework. For instance, political partisanship can directly affect policy acceptance, even when other aspects align with the desires of the target group, highlighting the importance of political consensus in policy acceptance. Adding this distinct component “Political Consensus,” our revised IPAC framework features at Figure 2.

 Regarding how religious leaders view international and geopolitical issues, our participants’ suspicion toward foreign-funded and produced vaccines due to their negative political perceptions of foreign donor organisations and countries also align with previous literature. Prior studies have found similar instances of vaccine hesitancy in various religious settings across different regions and for different vaccines, often driven by depopulation and anti-fertility conspiracy theories.^101-104^ Our participants were suspicious of anything “free” from Western and non-Muslim countries and organisations. They explained this through the lens of long-standing historical and political distrust and confrontations between Muslim and non-Muslim nations. Since the HPV vaccine is related to the female reproductive system and easily aligns with depopulation conspiracy, such rumours can spread quickly and demotivate people if not countered through policy interventions. Every time religious leaders expressed their concerns about the HPV vaccine, they consistently mentioned their fear of long-term side effects that could hinder fertility, aligning with prior literature identifying this same concern in other countries and communities.^4,103,105-108^ Recent studies have demonstrated the long-term safety and effectiveness of the HPV vaccine at reducing disease incidence and cervical cancer at the population level,^109-111^ and social influencers such as our participants (and hence the communities in which they operate) would benefit from wide dissemination of these findings. However, knowledge of scientific advancements alone will not address deep-seeded mistrust and doubt; amid ongoing political instability, this negative perception could flourish in the future.

###  Limitations of the Study

 This study has some limitations. Firstly, participants were grouped only by educational background and organizational affiliation, without considering geographical factors. Additionally, some participants had multiple educational backgrounds and affiliations, which might have influenced the findings. Secondly, this is a small study based on interviews with ten participants, which may not reflect the national context. However, since the study included participants from the major religious traditions/groups, these findings can be useful when considering how to engage religious leaders to support vaccine uptake. Thirdly, the more participants learned about HPV and its causes and prevention during the interview, the less supportive some became of the program. The interview format itself may have shaped participants’ responses. Discussions about HPV and STIs can be sensitive and responses can be influenced by how questions are framed, the order of discussion topics, the interviewer’s identity and disciplinary expertise, and perceived authority. Because we were interested to know how religious leaders would perceive and grapple with relationship between the vaccine, the disease and STIs, we did not shy away from presenting and discussing these issues. To mitigate negative responses, we tried to provide more information on HPV verbally after the interview and utilise awareness documents created by WHO and the government of Bangladesh. However, an approach that played down or did not engage with STIs may have obtained different results. These dynamics are common challenges in qualitative vaccine research globally, and future studies may benefit from incorporating trusted health workers as interviewers or utilising alternative elicitation techniques to minimise priming effects and improve participant comfort. Lastly, a major political movement led to the government’s overthrow, disrupting internet access and making some leaders hesitant to participate. Initially, 15 interviews were planned, but only 10 were conducted. More interviews could have provided deeper insights or alternative perspectives.

## Conclusion

 Bangladeshi religious leaders’ perceptions of cervical cancer, HPV, and STIs inform their acceptance of and support for the country’s vaccination program. Additionally, this study revealed that political polarisation and partisanship among religious leaders can affect health policy acceptance, either by not actively encouraging people or by actively demotivating them. When religious leaders perceive the government as opposing their political beliefs, their acceptance and compliance can decrease significantly.

 The IPAC framework is typically applied to public health and environmental policies in high income countries; this study applied it in a novel cultural context, examining the views of religious leaders as potential enablers rather than as end users. The framework helped identify how trust in government and the perceived intrusiveness of the policy shaped their stance. The study’s adaptation of IPAC to a religious, developing-world setting is an innovative extension, highlighting new factors such as political consensus.

## Acknowledgements

 The authors of this study express their gratitude to VaxPol Lab and its associated faculty members and staff for providing valuable insights during the study. They also extend their thanks to Samina Yasmeen, Emerita Professor at the School of Social Sciences, University of Western Australia, and the Centre for Muslim States and Societies. The authors are further grateful to Mr. A.S.M. Sanwar Rasel, Assistant Director, Department of Fisheries, Bangladesh; Mr. Abdul Aziz Bhuiyan, and Mr. Abhijit Chowdhury, Senior Assistant Secretary, Ministry of Public Administration, Bangladesh who work in the Bangladesh Civil Service in various capacities, for their assistance in reaching out to religious leaders for recruitment purposes. Additionally, they appreciate the participants who contributed their own perspectives as well as those who assisted with the snowball sampling process.

## Disclosure of artificial intelligence (AI) use

 Not applicable.

## Ethical issues

 Ethics approval for this study was granted by the Human Ethics & Clinical Trials Committee of the University of Western Australia on December 12, 2023 (Ref No: 2023/ET000954).

## Conflicts of interest

 Authors declare that they have no conflicts of interest.

## Disclaimers

 We confirm that this work is original and has not been published elsewhere, nor it is currently under consideration for publication elsewhere.
